# Secretome and Extracellular Vesicles as New Biological Therapies for Knee Osteoarthritis: A Systematic Review

**DOI:** 10.3390/jcm8111867

**Published:** 2019-11-04

**Authors:** Daniele D’Arrigo, Alice Roffi, Magali Cucchiarini, Matteo Moretti, Christian Candrian, Giuseppe Filardo

**Affiliations:** 1 Regenerative Medicine Technologies Laboratory, Ente Ospedaliero Cantonale (EOC), Via Tesserete 46, 6900 Lugano, SwitzerlandMatteo.Moretti@eoc.ch (M.M.); 2 Department of Biotechnology and Biosciences, University of Milano-Bicocca, Piazza dell’ Ateneo Nuovo 1, 20126 Milano, Italy; 3 Applied and Translational Research center (ATRc), IRCCS Istituto Ortopedico Rizzoli, Via di Barbiano 1/10, 40136 Bologna, Italy; ortho@gfilardo.com; 4 Center of Experimental Orthopaedics, Saarland University Medical Center, 66421 Homburg, Germany; mmcucchiarini@hotmail.com; 5 Cell and Tissue Engineering Laboratory, IRCCS Istituto Ortopedico Galeazzi, via Galeazzi, 4, 20161 Milan, Italy; 6 Orthopaedic and Traumatology Unit, Ospedale Regionale di Lugano, Ente Ospedaliero Cantonale, Via Tesserete 46, 6900 Lugano, Switzerland; Christian.Candrian@eoc.ch

**Keywords:** Exosome, extracellular vesicles, mesenchymal stem cell, knee osteoarthritis, injection

## Abstract

Secretome and extracellular vesicles (EVs) are considered a promising option to exploit mesenchymal stem cells’ (MSCs) properties to address knee osteoarthritis (OA). The aim of this systematic review was to analyze both the in vitro and in vivo literature, in order to understand the potential of secretome and EVs as a minimally invasive injective biological approach. A systematic review of the literature was performed on PubMed, Embase, and Web of Science databases up to 31 August 2019. Twenty studies were analyzed; nine in vitro, nine in vitro and in vivo, and two in vivo. The analysis showed an increasing interest in this emerging field, with overall positive findings. Promising in vitro results were documented in terms of enhanced cell proliferation, reduction of inflammation, and down-regulation of catabolic pathways while promoting anabolic processes. The positive in vitro findings were confirmed in vivo, with studies showing positive effects on cartilage, subchondral bone, and synovial tissues in both OA and osteochondral models. However, several aspects remain to be clarified, such as the different effects induced by EVs and secretome, which is the most suitable cell source and production protocol, and the identification of patients who may benefit more from this new biological approach for knee OA treatment.

## 1. Introduction

Osteoarthritis (OA) is a degenerative disease with progressive degradation of articular cartilage and subchondral bone leading to loss of joint function and pain which significantly impairs patient quality of life [1,2]. Worldwide estimates indicate that 9.6% of men and 18.0% of women over 60 years old suffer from symptoms of OA, with knee OA representing one of the most disabling conditions, with a huge social impact [3,4,5]. This high prevalence of OA is further increasing due to the augmented risk of OA due both to non-modifiable risk factors, such as the aging population and the gender, and to local risk factors, such as physical activity [6]. The classic clinical approaches in the treatment of OA offer mainly temporary symptom relief without disease modifying effects [7]. The limitations of available treatments fostered the development of new strategies, with cell-based procedures being proposed, such as minimally invasive injective approaches with the aim of modulating the inflammation process as well as stimulating and supporting the regeneration of articular tissues, thus re-establishing joint homeostasis. Mesenchymal stem cells (MSCs) represent the most promising cell population [8,9] showing, in several clinical studies, the possibility to increase joint function and reduce pain in knee OA patients [10,11,12]. However, the efficacy of this cell injection approach may be impaired by cell manipulation, and its wide application is strongly limited by regulatory issues [13,14].

To overcome these limitations, in the past 15 years researchers focused on the secretome of MSCs. In fact, it has been demonstrated that the therapeutic ability of MSCs is mainly related to their secretion of biologically active factors, rather than their differentiation properties [15]. These soluble factors belong to different biochemical classes and include growth factors, cytokines, chemokines, lipids, and other molecules with immunomodulatory effects [15]. All these paracrine factors, with the addition of a broad variety of acid nucleic and different lipids, can also be found within cell-secreted vesicles (extracellular vesicles (EVs)), a key part of the secretome which is gaining increasing attention by the scientific community. EVs (either microvesicles (MVs) or Exosomes (Exo)) represent important mediators between articular cell types [16]. Once secreted in the extracellular space, they can interact and be internalized by target cells, ultimately influencing and modifying their phenotype. Preclinical in vitro studies suggested a wide range of potential benefits with immunomodulatory, regenerative, anti-catabolic, and chondro-protective properties of secretome and EVs, which could overcome the limits of cell therapies while offering comparable biological effects. However, while secretome and EVs appear to be very promising, it is important to confirm their role and effects in the complex in vivo environment of knee OA joints [17].

Therefore, the aim of this systematic review was to analyze the available literature on both in vitro and in vivo settings, in order to understand the potential of secretome and EVs as a minimally invasive injective biological approach for the treatment of knee OA.

## 2. Materials and Methods

### 2.1. Data Source

A systematic review of the literature was performed on the use of secretome and EVs in both in vitro and in vivo studies for the treatment of OA affecting the knee joint. This search was performed on PubMed, Embase, and Web of Science databases up to the 31 August 2019 using the following string: (exosom* OR microvesicle* OR vesicle* OR ectosom* OR secretome) AND (mesenchymal stem cell* OR MSC* OR mesenchymal stromal cell* OR ASC* OR ADSC* OR BMC OR BMSC* OR stem cell*) AND (cartilage OR synovi* OR menisc* OR chondrocyte OR chondral OR osteoarthritis OR OA).

### 2.2. Study Selection Process

**Figure 1:**
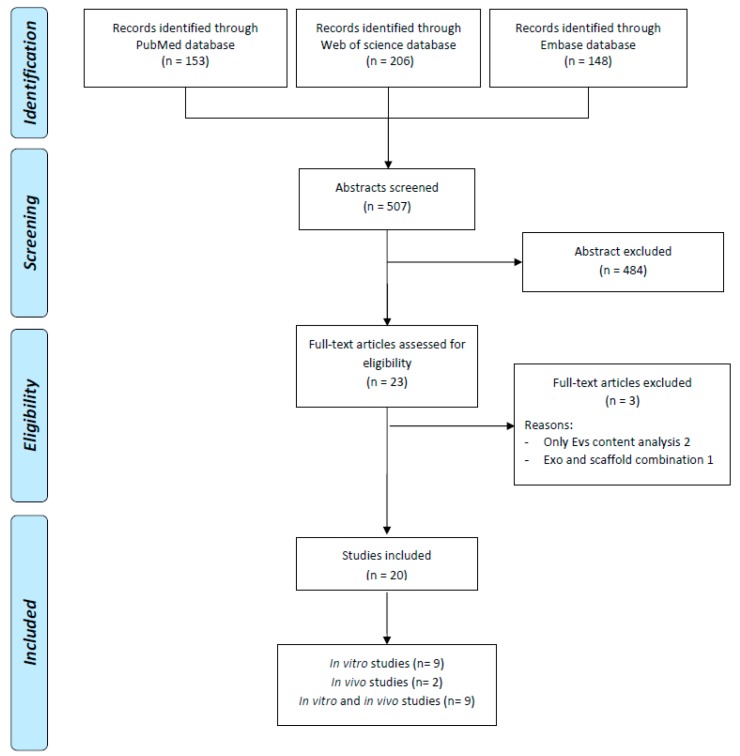
PRISMA (Preferred Reporting Items for Systematic Reviews and Meta-Analysis) flowchart of the systematic literature review.

Two independent reviewers (A.R. and D.D.) conducted the screening process and the analysis of the papers according to PRISMA guidelines. First, the reviewers screened the resulting records by title and abstract, then the full text of selected manuscripts was screened entirely according to the following inclusion criteria: in vitro and in vivo studies of any level of evidence, written in English language, on the use of secretome and EVs for the treatment of cartilage lesions and OA with focus on the knee joint. Exclusion criteria were articles written in other languages, reviews, studies not analyzing the effect of secretome and EVs or exploiting their potential effect not in the knee. The reviewers also screened the reference lists of the selected papers. The flowchart reported in Figure 1 graphically describes the systematic review process.

### 2.3. Data Extraction and Synthesis

**Figure 2:**
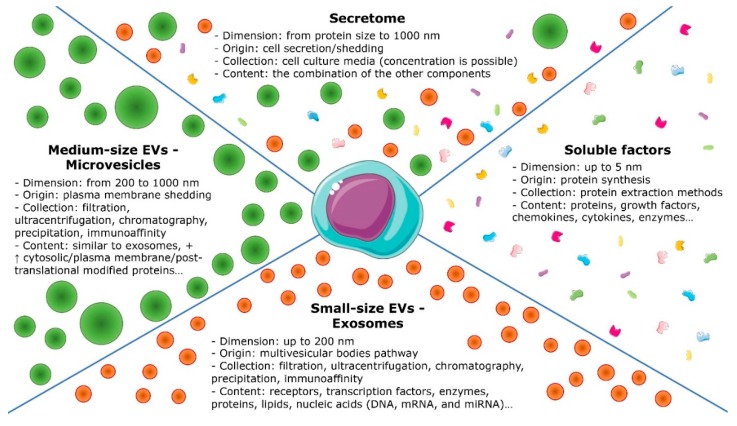
Schematic representation of secretome components.

From the included studies, relevant data were extracted, summarized, and analyzed according to the purpose of the present work. In particular, the following data were evaluated: cell source of secretome and EVs, target cell types, type of the secreted products (divided in secretome, Exo, and MVs, Figure 2), production method, storage, and study design; for the in vivo studies, the animal model was also considered together with the method of OA induction; in vitro effects were evaluated in terms of EVs internalization, effect on viability, proliferation and migration, effect on chondrocyte phenotype, production of cartilaginous ECM, anti-catabolic effect, anti-inflammatory and immunomodulatory effect, effect on apoptosis, autophagy, and senescence; the in vivo effects were evaluated in terms of effect on cartilage tissue and ECM deposition, effect on synovial inflammation and cytokines, effect on bone tissue, effect on pain, and gait.

## 3. Results

According to the search strategy, 154 papers were found from PubMed, 148 from Embase, 206 from Web of science. After duplicates removal, 20 papers were analyzed, nine of those were in vitro studies, nine were in vitro and in vivo, and two were in vivo studies. The in vitro studies have been described in detail in Table 1 while Table 2 pools together studies performed both in vitro and in vivo and those in vivo only. All these studies have been summarized in the following paragraphs.

### 3.1. In Vitro Studies

**Figure 3:**
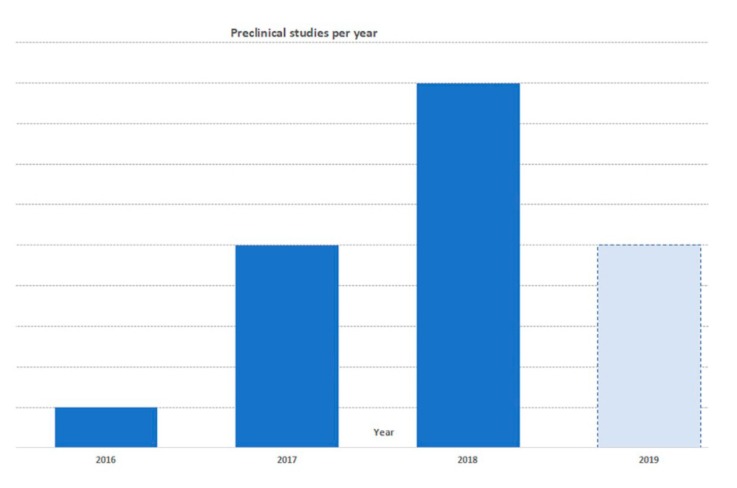
Published preclinical articles per year.

In vitro studies were published from 2017 with a rapidly increasing trend of publications (Figure 3). Among the 18 in vitro studies, six articles used bone marrow-derived MSCs (BMSCs), four adipose-derived stem cells (ASCs), two embryonic stem cell-derived MSCs (EMSCs), two commercial (not otherwise specified) MSCs, one synovial-derived MSCs (SMSCs), one chondrocytes, one infra patellar fat pad (IPFP)-derived MSCs, and one compared SMSCs with induced pluripotent stem cell line (iPSC)-derived MSCs. Furthermore, one study compared the effects of secretome, MVs, and Exo compared to BMSCs. Twelve articles investigated the effect of Exo, four evaluated Exo with MVs, one EVs (without other details), and one secretome. The most selected method to isolate EVs was differential centrifugation (five), followed by precipitation-based commercial kits (four), differential centrifugation coupled with a filtration step (three), filtration (three), differential centrifugation with sucrose density centrifugation (one), filtration and sucrose density centrifugation (one), while one paper did not report the detailed isolation protocol (one). Results of in vitro studies were summarized according to:

EVs internalization: EVs [18,19,20,21,22,23] can be internalized very quickly, already after 30 min from their administration [20]. Moreover, the kinetic of their uptake reached a maximum after 12–18 h [19,21] when cells appeared to be saturated, and continued up to the last evaluation performed at 24 h after EVs addition [19,21]. The intracytoplasmic localization of internalized Exo was identified in the perinuclear region [18,19,23].

Effect on viability, proliferation, and migration: A total of 14 papers investigated the effect of EVs or secretome on cell proliferation, viability, and migration (Table 1) [18,20,21,22,23,24,25,26,27,28,29,30,31,32]. Twelve papers reported that EVs derived from MSCs increased the proliferation and/or the viability of OA chondrocytes or chondrocyte progenitor cells [18,20,21,22,23,25,26,28,29,30,31,32], with a dose dependent effect [18,21,22,26], while two papers [24,27] reported no significant effects on chondrocytes viability. One study compared the effect on chondrocyte proliferation of Exo derived from two different MSCs sources [31], iPS-derived MSCs and SMSCs, finding that Exo from iPS-MSCs had superior effects than SMSC-Exo. While high proliferation mediated by SMSCs-Exo was correlated with a concomitant decrease of the extracellular matrix (ECM) components: this was not observed with Exo enriched with a particular micro RNA (miR-140-5p), as shown by Tao et al. [23]. Finally, seven articles [21,23,25,26,28,30,31] documented that cell migration increased after Exo administration, showing also dose dependency [21,26,28].

Effect on chondrocyte phenotype: Thirteen papers assessed phenotype maintenance and chondrocyte hypertrophy after the treatment with EVs or secretome [20,21,23,24,25,26,27,28,29,30,32,33,34]. Regarding the maintenance or the induction of chondrocyte phenotype, three studies described the increase of the expression of SOX9 [20,25,30], mediated by EVs. Six works analyzed chondrocyte hypertrophy status after EVs addition [20,24,28,29,30,32], and four of them showed that the expression of the transcription factor RUNX2 diminished with EVs [20,28,30,32]. Liu et al. [28] reported that this reduction was dose dependent. At protein level, four papers observed the decreased expression of type-X collagen mediated by EVs, either normal [20,24] or overexpressing miR-92a-3p [30] or miR-95-5p [29]. Similarly, the level of other two hypertrophy markers (alkaline phosphatase and osteocalcin) were lowered [20,24].

Production of cartilaginous ECM: ECM protein expression was investigated in 13 studies [19,20,21,23,25,26,27,28,29,30,32,33,34]. Among these papers, 11 quantified the expression of ECM components, in particular type-II collagen and aggrecan, in chondrocytes treated with EVs or secretome [20,23,25,26,27,28,29,30,32,33,34]. Nine papers reported induction of type-II collagen and aggrecan expression by EVs, with a dose dependent effect [28,33,34] and showed a tendency towards better effects exerted by MVs over Exo [27,33]. Only one study by Tao et al. [23] reported that SMSCs-Exo diminished the expression of COL2A1 and aggrecan in a dose-dependent manner, but this effect was reverted with the use of Exo overexpressing miR-140-5p. In general, all papers investigating the effects of EVs enriched with specific miRNA [25,26,29,30] reported a significant improvement of ECM protein level. Finally, Exo had positive effects also on Cartilage Oligomeric Matrix Protein (COMP) [21] and on HAS-1,2,3 levels [19] increase. In particular, Ragni et al. [19] showed that two days after Exo addition HAS-1 was up-regulated while HAS-3 was down-regulate and the isoform 2 did not show any significant variation.

Anti-catabolic effect: Twelve of the included papers evaluated the anti-catabolic effect of EVs on chondrocytes or synovial fibroblasts [19,20,24,25,26,27,28,29,30,32,33,34], showing a general positive effect with a dual action on catabolic proteins decrease or increase of their inhibitors. The expression of matrix metalloproteinase 13 (MMP-13) resulted lower in eight studies [19,26,27,28,29,30,32,33], with a dose-dependent effect [28]; while two papers did not observe any variation [24,25]. In particular, Sun et al. [25] demonstrated the superiority of Exo-miR-320c in decreasing MMP-13 levels with respect to normal Exo. Two works reported a tendency of superior effects exerted by MVs over Exo and secretome [27,33] in reducing MMP-13 expression [33] and MMP activity in a dose-dependent manner [27]. Ragni et al. [19] showed a reduction of MMP-1 levels at an early time point (2 days). On the other hand, Niada et al. [24] reported that secretome increased tissue inhibitors of MMPs (TIMP) -1, -2, -3, and -4, supporting that the minor MMP activity might be ascribed to the production of TIMPs. Three papers [26,33,35], investigated the expression of ADAMTS5, describing a comparable reduction in Exo and MVs groups, but greater than the one induced by the secretome [33]. Finally, Vonk et al. [20] showed that collagenase activity was significantly reduced by EVs administration, with a concomitant increase of Wnt-7a expression, which could contribute to the prevention of cartilage damage and to the regeneration process.

Anti-inflammatory and Immunomodulatory effect: Positive effects have also been reported for the anti-inflammatory and immunomodulatory action mediated by secretome or EVs, and described in six studies [19,20,27,30,33,36], showing the reduction of inflammatory mediators and the increase of anti-inflammatory molecules. In particular, it was demonstrated that the expression of Cyclooxygenase 2 (COX-2), Interleukin 1 alpha (IL-1α), -1 beta (β), -6, -8, and -17 [20], Tumor necrosis Factor alpha (TNF-α), IL-6, Microsomal Prostaglandin E Synthase-1 (mPGES-1), inducible Nitric Oxide Synthase (iNOS), and Prostaglandin E2 (PGE-2) [27] decreases following secretome or EVs administration, while anti-inflammatory factors like IL-10 increases [27]. Concerning the effects exerted by different EVs types, secretome was shown to be significantly more effective than Exo in decreasing COX-2 and mPGES-1 expression [27], as well as TNF-α quantity [33]. Conversely, the expression of iNOS showed a dose-dependent reduction following the administration of Exo and MVs, which both exerted significantly better results than the secretome [33]. Finally, the study of the polarization of macrophages phenotype showed that both Exo and MVs diminished their activation [33].

Effect on apoptosis: As increased chondrocyte apoptosis represents another feature of OA cartilage, six papers investigated the impact of EVs on this cell process [18,22,26,28,32,33], all reporting a significant decrease in apoptosis rate. Among these, three also demonstrated a dose dependent reduction of OA chondrocyte apoptosis [22,28,33], with superior results for Exo versus MVs [33]. One paper studied the effect of Exo overexpressing a long non-coding RNA (KLF3-AS1) [32], showing that not transfected MSC-Exo significantly reversed IL-1β-mediated chondrocyte apoptosis, and that KLF3-AS1-Exo consolidated this inhibition.

Autophagy and senescence: Another cell process important for cartilage biology during OA progression is autophagy, assessed by Wu et al. [26], showing that Exo significantly increased autophagy in IL-1β-treated chondrocytes. Finally, Tofiño-Viann et al. [36] demonstrated that Exo, MVs, and secretome significantly reverted mitochondrial membrane increase and oxidative stress induced by IL-1β, thus causing a reduction in DNA damage and resulting in inhibition of the senescence process.

### 3.2. In Vivo Studies

In vivo studies were published from 2016 with a rapidly increasing trend of publications (Figure 3). Among 11 in vivo studies, nine included both an in vitro investigation and an animal model study. Six studies have been performed in mouse, four in rat, and four in rabbit. Three studies created an osteochondral defect model and eight an OA model. Eight articles investigated the effect of Exo, 1 of secretome, one of MVs, and one compared Exo with MVs. Regarding the cell source, four used EVs or secretome from BMSCs, three from EMSCs, two from SMSCs, one from IPFP, and one commercial not better specified MSCs. The most selected method to isolate EVs was differential centrifugation (four), followed by ultrafiltration (two), filtration (two), precipitation-based commercial kits (one), and sucrose density centrifugation (one). All studies showed positive effects after the administration of secretome, Exo, or MVs in both osteochondral [21,22,35] and OA defect models [23,26,28,30,31,33,34,37]. The results of in vivo studies have been summarized according to:

Effect on cartilage tissue and ECM deposition: Animal studies showed that Exo was effective in cartilage surface restoration and ECM deposition [21,26,31,34,35], regenerating a hyaline-like cartilage completely integrated with the adjacent tissues [31,35]. Zhang et al. [21] demonstrated that this repair and the deposition of ECM started 2 weeks post-injection and increased over time for up to 12 weeks. Similar results after Exo and MVs injections were reported, both providing protection from OA development [33] and showing that both vesicles are equally effective in counteracting tissues degeneration and promoting cartilage regeneration. Positive effects on cartilage repair and ECM deposition have also been described for Exo derived from cells over-expressing microRNA [23,30] or engineered to silence specific genes [28]. These results were superior to those induced by normal Exo. Finally, Khatab et al. [37] and Xiang et al. [22] demonstrated that the effect of secretome and MVs injections on cartilage and ECM were the same as those exerted by MSC injection.

Effect on synovial inflammation and cytokines: Two studies addressed this issue, one showing that Exo increased M2 macrophage infiltration while decreased M1 and inflammatory cytokines [21], while the other study was unable to demonstrate any effect on synovial inflammation for both secretome and MSCs [37].

Effect on bone tissue: Regarding subchondral bone, both Exo and MVs were effective in terms of regeneration: Zhang et al. [21,35] showed complete subchondral bone restoration; Cosenza et al. [33] described higher bone volume and lower bone degradation at epiphyseal and subchondral level following MVs or MSCs injections with respect to controls. Conversely, no effect on bone remodeling was reported by Khatab et al. for both secretome and MSCs [37].

Effect on pain and gait: Another interesting aspect is that Exo injections were able to partially ameliorate gait abnormality patterns in the OA mouse model [26]. Moreover, Khatab et al. [37] demonstrated that both secretome and MSCs provided early (day 7) pain reduction in the treated animals.

## 4. Discussion

The main finding of this systematic review is that the use of secretome and EVs for the treatment of cartilage pathology and knee OA had pleiotropic effects and overall positive results. In vitro, both secretome and EVs showed anticatabolic, immunomodulatory, and regenerative properties, and in vivo studies confirmed the effectiveness as minimally invasive treatment, with positive effects on the whole joint.

The literature analysis supports the use of secretome and EVs with an increasing number of preclinical studies. The overall successful results, coupled with the same low immunogenicity of MSCs, and potentially fewer legal issues compared to therapies based on cell transplantation [38], make this biological approach a good candidate for human translatability. The use of secretome and EVs as a minimally invasive treatment for OA in an in vivo preclinical model showed that it was equally effective as MSCs in terms of pain improvement and morphological changes [37], and even proved the superiority of MVs and Exo over BMSCs in terms of joint protection from OA [33]. On the other hand, the literature analysis also underlined that, despite the increasing interest with many recent publications, this field is still in its infancy, with several approaches proposed but lacking the underlying understanding of biological roles and functions. In addition, standardizations and indications on the most suitable strategies for exploiting the potential of this biological approach are also still lacking.

With the aim to evaluate the potential of secretome and EVs as new cell derived approaches for the treatment of knee OA, the available literature was screened for both in vitro and in vivo studies assessing the role of these biological products in the different physiologic processes involved in cartilage lesions and OA progression and treatment. Three different cell derived products were considered: secretome, Exo, and MVs. For this analysis, the secretome group included all studies that specifically referred to the secretome. Regarding the EVs, they are a heterogeneous population which has been classified into three classes according to their biogenesis and size: apoptotic bodies, MVs, and Exo [39]. The apoptotic bodies, the largest EVs population, range from 200 nm to 5000 nm, and they are secreted by the shedding of the plasma membrane of apoptotic dying cells. The MVs, also called ectosomes or microparticles, are 200–800 nm sized EVs that are shed from the plasma membrane of viable cells. Exo, which are 30–200 nm in size, are formed intracellularly and then released within the multivesicular bodies pathway. However, this classic EVs nomenclature results overburdened and sometimes confusing [40]. For the purpose of this systematic review EVs have been subdivided in two different population as small (below 200 nm) and medium-sized EVs (larger vesicles), following the statement of the of the International Society for Extracellular Vesicles [41], but maintaining the nomenclature used in all the papers analyzed, Exo and MVs respectively.

The literature analysis showed a great heterogeneity among studies in terms of EVs used, size, and isolation procedures. The most investigated EVs type is Exo, with a different size range (from 50 to 200 nm), making it difficult to compare among studies or correlate EVs characteristics and in vitro and in vivo results. Only one study [33] investigated the effect of different EVs types, comparing MVs and Exo on a chondrocytes culture and an in vivo OA model. The study showed that both EVs exert similar chondroprotective and anti-inflammatory effects, delaying OA development, leaving the question on the most suitable approach still open. The isolation procedures represent a critical aspect, since there is no standardized method to isolate EVs, resulting in different protocols and therefore different products to be used. The main methods used are differential centrifugation, filtration, and precipitation-based reagent, but there is a lack of standardized methods to obtain them, possibly contributing to EVs variability. Moreover, secretome and EVs can be obtained from different cell sources. 

This systematic review showed that the most used cell source are currently BMSCs, followed by ASCs, EMSCs, SMSCs, but there is lack of information available about the difference between vesicles derived from different cells and thus the optimal cell source to address OA remains elusive. Only one study [31] compared the effects of Exo secreted by iPS-derived MSCs and SMSCs in vitro. This showed that they both stimulated chondrocyte proliferation in a dose-dependent manner, but results depended on the cell source, with superior effect of Exo from iPS-derived MSCs on cell proliferation, at high concentration, and superior therapeutic effect in attenuating OA in a mouse model. 

The proper selection of EVs cell source and also the stage of cell differentiation are actually critical aspects, since they can determine the characteristics and properties of EVs to fit specific applications (such as reducing inflammation, promoting cartilage regeneration and protection from OA features) [38]. Furthermore, the surrounding microenvironment seems to play an active role in determining the composition of both secretome and EVs cargo, ultimately affecting their action on target cells [42]. Analogously, the type of media and substrate used for cell culture, as well as the use of primary or immortalized cells can also independently affect secretome and EVs composition [38]. For the translational potential of secretome and EVs into a clinically available therapeutic option, another key factor to be considered is the proper dosage [21,22,29,30,33]. In this regard, the literature presents concordant findings, with all papers that compared different amounts reporting a dose dependent effect and superior results at the higher quantities. However, no effects of different dosages were described in the analyzed animal models. In addition, the lack of standardization, also in terms of unit of measurement employed to express the used amount of EVs and thus the presence of heterogeneous products, prevents the possibility to identify the best EVs concentration for an optimal effect in terms of OA treatment. Further efforts should investigate the protocols to optimize secretome and EVs production toward OA treatment.

While studies focusing on the most suitable cell source and dosage could foster the clinical translatability of this biological approach, research efforts are already invested into the investigation on how to further develop this field by optimizing secretome and EVS potential. In this light, among the beneficial effects mediated by EVs, one aspect remains critical: Tao et al. [23] reported that chondrocytes treated with normal Exo decreased ECM proteins expression. On the contrary, the treatment with miR-140-5p-Exo, expressed during the development and homeostasis of cartilage and lowered in OA [43], did not affect ECM protein secretion. miRNAs are important Exo components and their role has been demonstrated in repressing chondrocytes inflammation, promoting chondrogenesis, and inhibiting cartilage degeneration. Considering these effects on chondrocytes, five studies [23,25,26,29,30] investigated the overexpression of different miRNAs in Exo, describing in general better results compared to the normal Exo in terms of cell proliferation, gene expression, ECM components’ production in vitro and inhibition of cartilage degradation in vivo. Furthermore, Liu et al. [32] described the effect of over expressing a long non-coding RNA KLF3-AS1, a competitive endogenous RNA which was able to inhibit miR-206, a miRNA that resulted overexpressed in OA.

Another feature of OA is synovial inflammation, notably characterized by activation of monocytes and macrophages. One major immunosuppressive effect of BM-MSCs is to inhibit macrophage activation and to induce a shift from M1 pro-inflammatory to M2 anti-inflammatory phenotype [44]. In this light, Cosenza et al. [33] demonstrated that both MVs, Exo, and BM-MSCs inhibited in vitro macrophage activation to a similar extent. On the contrary, Khatab et al. [37] did not report any significant change on synovial thickness or synovial macrophages phenotypes using secretome injection in an OA mouse model, although several significant moderate correlations between macrophage phenotypes and OA characteristics were found. Another aspect was investigated: different types of stress can lead to a premature cellular senescence. Among these, chronic inflammation can increases oxidative stress driving to cellular senescence, a process that can contribute to the development and progression of OA [45]. In this context, Exo was able to revert the oxidative stress induced by IL-1β, thus causing a reduction in DNA damage and resulting in inhibition of the senescence process [36].

All these investigated targets confirmed the pleiotropic effects of secretome and EVs, which led to positive effects also in vivo. Exo injections were able to partially ameliorate the gait abnormality patterns in the OA mouse model [26], and secretome injections provided early (day seven) pain reduction in treated animals, similar to MSCs [37], further supporting the translational potential of this biological approach. On the other hand, this systematic review also underlined several critical aspects needing additional investigation to further develop and optimize this biological treatment strategy. The promising in vitro and in vivo results support the potential of this new treatment approach, opening new perspectives for cell-based therapies. Secretome and EVs could require less complex regulation procedures than treatments based on cell transplantation, while providing similar results of MSCs. The standardization of protocols could further facilitate clinical translatability. In this light, research efforts are required for the identification of the proper cell source, the best preparation protocol and the most suitable target and, in the end, for the translation of the preclinical promising findings into clinical trials to confirm the potential of secretome and EVs as a minimally invasive biological treatment to address knee OA.

## 5. Conclusions

This systematic review of the literature underlined an increasing interest towards this emerging field, with overall positive findings. Promising in vitro results have been documented in terms of enhanced cell proliferation, reduction of inflammation, and down-regulation of catabolic pathways while promoting anabolic processes. The positive in vitro findings were confirmed in vivo, with studies showing positive effects on cartilage, subchondral bone, and synovial tissues in both OA and osteochondral models. However, several aspects remain to be clarified, like the different effects induced by EVs and secretome, the most suitable cell source and production protocol, as well as the identification of patients that may benefit more from this new biological approach for the treatment of knee OA.

## Figures and Tables

**Table 1:** Details of papers with in vitro experiments. Abbreviations: hBMSCs, human bone marrow-derived mesenchymal stem cells; Exo, exosome; EV, extracellular vesicles; HA, hyaluronic acid; ASC, adipose-derived mesenchymal stem cells; MVs, microvesicles; CM, conditioned medium; FLS, fibroblast-like synoviocytes.

References	Cell types	Secretome/Vesicle Types	Isolation Method and Storage	Study Design	Results
Niada et al. 2019 Stem Cell Res	Source: Human ASCs (2 males + 6 females; mean age: 46 ± 16 years) Target: Human chondrocytes (11 males + 7 females; mean age: 62 ± 11 years)	Secretome	Cell Passage: NA Secretome collected after 72 h of ASCs starvation (ca 10^6^ ASCs)	CH + TNFa vs. CH + TNFa + ASCs-secretome	No alteration on chondrocytes viability. Secretome addiction reduce osteocalcin, collagen X (anti-hypertrophic effect) MMP3, 13 activity reduction correlated to the abundance of TIMPs in secretome (anticatabolic effect)
Sun et al. 2019 J Cell Biochem	Source: Human BMSCs (3 males; mean age: 27, 3 years) Target: Human chondrocytes (3 males; mean age: 68.2 ± 7.1 years)	Exo (50–200 nm)	Cell passage: 3 Isolation method: Precipitation Storage at −80 °C	hBMSC-320c-Exos vs. hBMSC-Exo vs. PBS	Increase of chondrocyte proliferation in hBMSC-320c-Exo group than other Exo, with down-regulated MMP 13 and up-regulated Sox 9 expression during hBMSC chondrogenic differentiation.
Ragni et al. 2019 Stem Cell Res & Ther	Source: Human ASCs (3 females; mean age: 54 ± 8 years) Target: Human fibroblast-like synoviocytes (3 females; mean age: 72 ± 7 years)	EVs (40–400 nm)	Cell passage: range 3–5 Isolation method: Differential centrifugation Storage: at 4 °C if used within 2 days or −80 °C	EVs vs. EVs + HA	HA is involved in EV internalization EVs reduced the expression of pro-inflammatory cytokines and chemokines in a chronic model of FLS inflammation
Qi et al. 2019 In vitro cell & develop biology	Source: Rabbit BMSCs (males; age: 4 weeks) Target: Rabbit chondrocytes (males; age: 4 weeks)	Exo (50–150 nm)	Cell passage: 3 Isolation method: Filtration + Ultracentrifugation Storage: NA	BMSCs Exo vs. control	All changes induced by Il1b, as decreased cell viability, increased apoptosis was abolished by the addition of BMSCs-Exo.
Liu et al. 2018 Cell Cycle	Source: Commercial MSCs Target: Mouse chondrocytes	Exo (NA)	Cell passage: 3 Isolation method: Exo isolation reagent Storage: NA	MSCs vs. MSCs-Exo	MSC-Exo increased chondrogenic genes Col2a1 and aggrecan, decreased MMP-13 and Runx2. Moreover, MSC-Exo induced cells proliferation and cells apoptosis inhibition
Mao et al. 2018 J Cell Mol Med	Source: Human miR-95-5p- chondrocytes (3 males + 3 females; mean age: 35 years) Target: Human chondrocytes (3 males + 3 females; mean age: 53, 6 years)	Exo (90–200 nm)	Cell passage: 3 Isolation method: Differential centrifugation Storage NA	AC-miR-95-5p-Exo vs. AC Exo (10 µg vs. 50 µg)	50 μg Exo/mL AC-miR-95-5p-Exo showed greater proliferation than those incubated with other doses. Up-regulated the expression levels of aggrecan, COL2A1, COL9A1, COMP. Decrease COL10A1, MMP13, HDAC2-8
Vonk et al. 2018 Theranostic	Source: Human BMSCs (1 male + 1 female; mean age: NA) Target: Human chondrocytes (5 female + 3 male; mean age: NA)	EVs (40–150 nm)	Cell passage: BMSCs 4–7, Chondrocytes 2 Isolation method: Ultracentrifugation + sucrose density Storage NA	EVs vs. CM vs. CM-EVs	BMMSC-EVs down-regulated COX-2, pro-inflammatory interleukins and inhibited TNF-alpha-induced collagenase activity. Increase proteoglycans production and type II collagen
Tofiño-Vian et al. 2018 Cell Physiol Biochem	Source: Human ASCs (4 males + 7 females; mean age 53, 8 years) Target: Human chondrocytes (27 females + 14 males; mean age: 65, 6 years)	MVs (mean 279 nm) and Exo (mean 104 nm)	Cell passage: 0 Isolation method: Filtration + Ultracentrifugation Storage: −80 °C	MVs vs. Exo vs. CM vs. control	MVs and Exo reduced the production of inflammatory mediators (TNFα, IL-6, PGE_2_, NO) Anti-inflammatory and chondroprotective effect mediated by up-regulation of annexin1, especially in MVs group
Tofiño-Vian et al. 2017 Oxid Med Cell Longev	Source: Human ASCs (2 males + 2 females, mean age 54, 4 years) Target: Human osteoblasts (21 females + 9 males; mean age: 68, 4 years)	MVs (mean 316 nm) and Exo (mean 115 nm)	Cell passage: 0 Isolation method: Filtration + Ultracentrifugation Storage: −80 °C	MVs vs. Exo vs. CM vs. control	CM, MVs, and Exo down-regulate senescence activity and reduced the production of inflammatory mediators

**Table 2:** Details of studies with in vitro and in vivo experiments. Abbreviations: IPFP, infrapatellar fat pad MSCs; I.A., intra-articular; Exo, Exosome; BMSCs, bone marrow-derived MSCs; MVs, microvesicles/microparticles; SMSC, synovial MSCs; ACs, articular chondrocytes; ES, embryonic stem cells; CM, conditioned medium; iMSC, induced pluripotent stem cell-derived MSCs; ECM, extracellular matrix.

References	Animal and OA Model	Sources	Secretome/Vesicle Types	Isolation Method and Storage	Study Design	Results
**In vivo studies**						
Khatab S et al. 2018 ECM Journal	Mouse Collagenase-induced OA model	Human BMSCs (female:male ratio: 1:2; mean age 55.3 ± 10 years)	Secretome	Cell Passage: 3 Storage: −80 °C	MSCs secretome vs. MSCs vs. medium	Pain reduction at day seven and better cartilage repair for MSCs secretome and MSC-groups compared to the control. No effects on synovial inflammation, subchondral bone volume or presence of different macrophage subtypes
Zhang et al. 2016 Osteoarthritis and Cartilage	Rat Surgical-osteochondral defect model	Human embryonic stem cell-derived MSCs	Exo (mean 100 nm)	Cell passage: NA Isolation method: FiltrationStorage at −20 °C	Exo (100 mg) vs. PBS	In Exo group, complete restoration of hyaline-like cartilage and subchondral bone with good surface regularity, complete bonding to adjacent cartilage, and ECM deposition
**In vitro and in vivo studies**						
Wu J et al. 2019 Biomaterials	Mouse Surgical meniscus destabilization OA model	Source: Human IPFP MSCs Target: Human chondrocytes	Exo (mean 121.9 nm)	Cell Passage: 1 Isolation method: Ultrafiltration Storage: −80 °C	In vitro: IPFP-MSCs vs. Exo In vivo study 1 sham vs. PBS vs. Exo-IPFP In vivo study 2 PBS + antagomir-NC vs. PBS-ExoIPFP + antagomir-NC vs. Exo-IPFP+ antagomir-100-5p	In vitro: Cell apoptosis inhibition, matrix synthesis promotion, reduction of catabolic markers expression, enhance autophagy level via mTOR inhibition In vivo: Exo significantly prevent cartilage destruction and partially improve gait abnormality. I.A. of antagomir demolishes the remedial effect of MSCs Exo
Liu et al. 2018 Biochem J	Rat Collagenase-induced OA model	Source: Commercial MSCs Target: mouse chondrocytes	Exo (NA)	Cell passage: NA Isolation method: isolation reagent kit Storage: NA	In vitro: Exo vs. Exo- KLF3-AS1 In vivo Normal vs. OA vs. PBS vs. MSCs-Exo vs. MSCsi-KLF3-AS1-Exo	In vitro: Exo KLF3-AS1 suppressed IL-1β-induced apoptosis of chondrocytes and promote chondrocytes proliferation.In vivo: Exo-KLF3-AS1 promoted cartilage repair in an OA rat model.
Mao G et al. 2018 Stem Cell Res & Ther	Mouse Collagenase-induced OA model	Source: Human BMSCs (3 males + 3 females; mean age: 35 years) Target: OA Primary human chondrocytes (3 males + 3 females; mean age: 60.24 years) Normal human chondrocytes (3 males + 3 female; mean age: 54.46 years)	Exo (50–150 nm)	Cell passage: BMSCs 3; primary chondrocytes 0 Isolation method: Differential Centrifugation Storage: NA	In vitro: Exo (50, 100, and 200 µg) vs. miR-92a-3p-Exo (50, 100, and 200 µg) vs. PBS for proliferation For other tests 200 µg In vivo: MSC-Exo (500 μg/mL) vs. MSC-miR-92a-3p-Exo (500 μg/mL) vs. OA and normal groups	In vitro: miR-92a-3p- Exo promoted higher cell proliferation, matrix genes expression in MSCs and chondrocytes, respectively at 200 ug concentration In vivo: MSC-miR-92a-3p-Exo inhibited cartilage degradation in OA model
Xiang et al. 2018 Transl Res	Rabbit Surgical osteochondral defect model	Source: Human BMSCs (commercial) Target: Human chondrocytes (commercial)	MVs (mean 200 nm)	Cell passage: NA Isolation method: Ultrafiltration + sucrose cushion Storage: NA	In vitro: MVs 5 µL vs. MVs 10 µL vs. MVs 20 µL vs. PBS In vivo: 3 weekly I.A. injection PBS vs. BMSCs vs. MVs	In vitro: MVs induced chondrocytes proliferation in a dose dependent manner and protected chondrocytes from apoptosis via bioactive lipid, S1P In vivo: MVs significantly accelerated cartilage recovery as BMSCs. Blocking S1P in vivo reduced the therapeutic effect of MVs
Zhang et al. 2018 Biomaterials	Rat Surgical osteochondral defect model	Source: Human immortalized embryonic stem cell-derived MSCs Target: Rat chondrocytes (age: 8 weeks)	Exo (mean 100 nm)	Cell passage: 2 Isolation method: Filtration Storage at −20 °C	In vitro: Control vs. Exo 1 µg/mL vs. 5 ug/mL vs. 10 µg/mL In vivo: Exo (100 mg) vs. PBS (100 mL)	In vitro: Exo groups induced cells proliferation and migration increase, with dose-dependent effect; matrix synthesis increase; apoptosis decrease In vivo: In Exo group, initial repair at 2 weeks with neotissue formation and ECM deposition. Improved surface regularity and integration at 6 weeks and complete integration at 12 weeks. MSC Exo increase M2 macrophage infiltration with a concomitant decrease in M1 macrophages and inflammatory cytokines
Cosenza et al. 2017 Scientific Report	Mouse Collagenase-induced OA model	Source: Mouse BMSCs Target: Mouse Chondrocytes (age: 3 days) Mouse macrophages (age: 3 days)	Exo (mean 112 ± 6.6 nm) and MVs (mean 223 ± 15.6 nm)	Cell passage: BMSCs 10–20; Isolation method: Centrifugation No storage, freshly use	In vitro: MVs or Exo (12.5 ng; 125 ng or 1.25 μg) vs. BM-MSC-CM (1 mL) vs. BM-MSCs (10^5^ cells). In vivo: a single I.A. injection BM-MSCs (2.5 × 10^5^ cells/5 μL saline) vs. MVs (500 ng/5 μL) vs. Exo (250 ng/5 μL)	In vitro: MVs and Exo exerted similar chondroprotective and anti-inflammatory function In vivo: MVs and Exo protected from developing OA
Tao et al. 2017 Theranostic	Rat Surgical meniscus destabilization OA model	Source: Human SMSC Target: Human Chondrocytes (age: 45–55 years)	Exo (30–150 nm)	Cell passage: SMSC 5 Isolation method: Centrifugation No storage, freshly use	In vitro: SMSC-Exo vs. SMSC-140-Exo In vivo: 4 weekly I.A injection sham vs. saline vs. SMSC-140-Exo vs. SMSC-Exo	In vitro: SMSC-140-Exo enhanced the proliferation and migration of ACs without damaging ECM secretion In vivo: SMSC-140-Exo slowed the progression of early OA and prevent severe damage to knee articular cartilage.
Wang et al. 2017 Stem Cell Research & Therapy	Mouse Surgical meniscus destabilization OA model	Source: Human ES embryonic stem cells (females, commercial) Target: Mouse chondrocytes (age: within 2 days)	Exo (38 nm to 169 nm)	Cell passage: ES 4–7; chondrocytes 3 Isolation method: Centrifugation Storage at −80 °C	In vitro: CM+Exo vs. CM vs. Exo In vivo: 2 I.A. injections/week Exo (1 × 10^6^) vs. PBS	In vitro: Chondrocyte phenotype maintenance in Exo group by increasing collagen type II synthesis and decreasing ADAMTS5 expression In vivo: Exo alleviated cartilage destruction and matrix degradation
Zhu et al. 2017 Stem Cell Research & Therapy	Mouse Collagenase-induced OA model	Source: Human SMSC (2 males + 1 female; age: 22–28 years); iPS-derived MSCs (iMSC, commercial) Target: Human chondrocytes	Exo (50–150 nm)	Cell passage: NA Isolation method: Ultrafiltration Storage at −80 °C	In vitro: iMSC-Exo vs. SMSC -Exo In vivo: 3 weekly I.A. injection normal vs. iMSC-Exo (1.0 × 10^10^/mL) vs. SMSC -Exo (1.0 × 10^10^/mL) vs. untreated	In vitro: Chondrocyte migration and proliferation were stimulated by both iMSC-Exo and SMMSC-Exo, with iMSC-Exo exerting a stronger effect. In vivo: iMSC-Exo and SMMSC-Exo both attenuated OA in the mouse OA model, but iMSC-Exo had a superior therapeutic effect compared with SMMSC-Exo.
